# Expression of *RsPORB* Is Associated with Radish Root Color

**DOI:** 10.3390/plants12112214

**Published:** 2023-06-03

**Authors:** Da-Hye Kim, Sun-Hyung Lim, Jong-Yeol Lee

**Affiliations:** 1 Division of Horticultural Biotechnology, School of Biotechnology, Hankyong National University, Anseong 17579, Republic of Korea; 2 Research Institute of International Technology and Information, Hankyong National University, Anseong 17579, Republic of Korea; 3 National Institute of Agricultural Sciences, Rural Development Administration, Jeonju 54874, Republic of Korea

**Keywords:** chlorophyll biosynthesis, green radish, *RsPORB*, polymorphisms, promoter variation

## Abstract

Radish (*Raphanus sativus*) plants exhibit varied root colors due to the accumulation of chlorophylls and anthocyanins compounds that are beneficial for both human health and visual quality. The mechanisms of chlorophyll biosynthesis have been extensively studied in foliar tissues but remain largely unknown in other tissues. In this study, we examined the role of NADPH:protochlorophyllide oxidoreductases (PORs), which are key enzymes in chlorophyll biosynthesis, in radish roots. The transcript level of *RsPORB* was abundantly expressed in green roots and positively correlated with chlorophyll content in radish roots. Sequences of the *RsPORB* coding region were identical between white (948) and green (847) radish breeding lines. Additionally, virus-induced gene silencing assay with *RsPORB* exhibited reduced chlorophyll contents, verifying that *RsPORB* is a functional enzyme for chlorophyll biosynthesis. Sequence comparison of *RsPORB* promoters from white and green radishes showed several insertions and deletions (InDels) and single-nucleotide polymorphisms. Promoter activation assays using radish root protoplasts verified that InDels of the *RsPORB* promoter contribute to its expression level. These results suggested that *RsPORB* is one of the key genes underlying chlorophyll biosynthesis and green coloration in non-foliar tissues, such as roots.

## 1. Introduction

**Figure 1:**
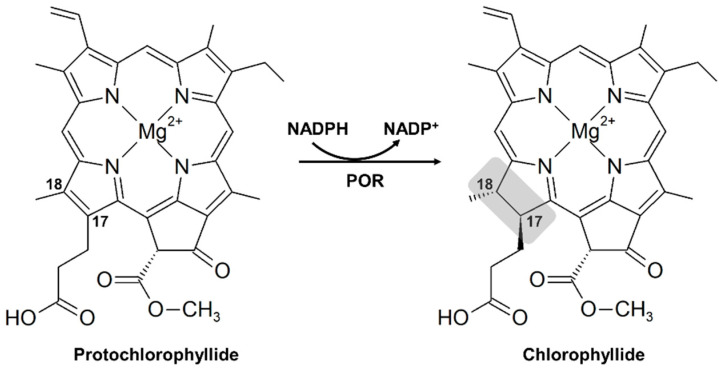
NADPH:protochlorophyllide oxidoreductase (POR) enzymes convert protochlorophyllide (Pchlide) into chlorophyllide (Chlide) by reducing the double bond between C17 and C18, using NADPH as the H donor. The gray box indicates the single bond resulting from the reduction.

Chlorophyll, one of the most abundant pigments on Earth, absorbs light energy from the sun and initiates the photosynthetic electron transfer system that results in ATP and NADPH production for energy and reducing power. However, excessive amounts of activated chlorophyll or chlorophyll intermediates can generate reactive oxygen species, damaging biomolecules and interfering with plant growth and development [1,2]. Therefore, the expression of genes involved in chlorophyll biosynthesis and degradation is tightly regulated with regard to specific organs, developmental stages, and environmental factors [3]. NADPH:protochlorophyllide (Pchlide) oxidoreductase (POR) is an essential enzyme that converts Pchlide into chlorophyllide (Chlide) by catalyzing the *trans* addition of hydrogen across the C17-C18 double bond of Pchlide, a critical intermediate metabolite in chlorophyll synthesis (Figure 1) [4,5]. In the dark, PORs, NADPH, and Pchlide form a ternary complex, the main component of the prolamellar body (PLB) in the etioplast. Under illumination, the PLB in the etioplast collapses and thylakoids emerge, resulting in chlorophyll accumulation and the formation of functional chloroplasts [4,6].

Several studies have revealed that *POR* genes encode highly conserved polypeptides and are ubiquitous in oxygenic photosynthetic organisms such as cyanobacteria, mosses, gymnosperms, and angiosperms [4]. The number of *POR* genes in an organism varies depending on the species. Among angiosperms, barley (*Hordeum vulgare*) has two *POR* genes, *HvPORA* and *HvPORB* [7], and *Arabidopsis (Arabidopsis thaliana*) has three, *AtPORA*, *AtPORB*, and *AtPORC*, which show different expression profiles [8,9]. In dark-grown barley seedlings, *HvPORA* mRNAs are abundantly detected, but they are strongly downregulated by illumination. In contrast, *HvPORB* transcripts are abundant regardless of light conditions [10]. For *AtPORA*, both transcripts and protein are detected in etiolated seedlings but are dramatically downregulated by illumination. In contrast, *AtPORB* transcripts and protein are present in both etiolated seedlings and light-grown plants, whereas those of *AtPORC* are upregulated in the light and downregulated in the dark [4]. These results indicate that the activities and amounts of POR proteins are regulated by light.

Radish (*Raphanus sativus*), a member of the Brassicaceae family, is an economically important vegetable crop widely cultivated for its roots, seed oil, and young sprouts [11]. Root color is an important trait of radish, along with shape and taste. The absence and/or presence of chlorophyll and anthocyanin pigments confers a variety of colors to radish roots, including white, green, red, and purple as well as bicolor patterns, e.g., white and red or white and green [12,13]. Comparative transcriptomic analysis with differently colored radish cultivars indicated that chlorophyll biosynthetic genes, including *POR*, *Chlide a oxygenase*, and *7-hydroxymethyl chlorophyll a reductase*, are significantly upregulated during the greening process in roots [14,15]. These studies revealed that green flesh with chlorophyll accumulation showed a high expression level of chlorophyll biosynthesis genes, but a low level of chlorophyll degradation genes. However, detailed molecular mechanisms of the expression level of chlorophyll biosynthetic and degradation genes in non-foliar tissues remain unknown.

To address this lack of information, we investigated the mechanism of chlorophyll biosynthesis in radishes with differently colored roots. Expression analyses revealed that the transcript level of *RsPORB* very well correlated with root color and chlorophyll content. Sequence analysis revealed nucleotide variation in the *RsPORB* promoter region, but not in the coding region. We analyzed *RsPORB* expression levels using both native and mutated *RsPORB* promoter constructs and confirmed that *cis* elements are important determinants of its expression level. This study is thus an important first step in elucidating the biosynthetic mechanisms underlying chlorophyll synthesis in non-foliar tissues.

## 2. Results

### 2.1. Chlorophyll Accumulation Determines the Skin and Flesh Color of Green Radish Roots

**Figure 2:**
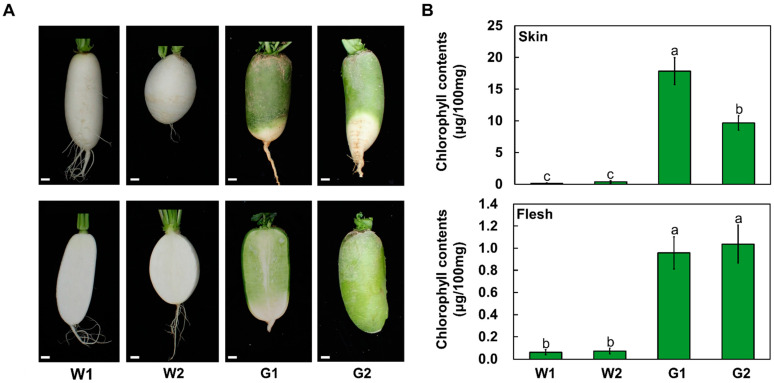
Phenotypes and chlorophyll content of root skin and flesh of four different radishes. (**A**) Root skin (top) and root flesh (bottom) of representative samples of two white (W1, W2) and two green (G1, G2) radishes. Bar = 1 cm. (**B**) Chlorophyll contents in root skin and root flesh. Results represent the mean values ± SD from three independent experiments. Different letters indicate significantly different values (*p* < 0.05), as determined using a one-way ANOVA followed by Duncan’s multiple range tests.

To observe root phenotypes, we cultivated four different radishes in the field for 8 weeks after sowing (Figure 2A). The two white root radishes, ‘948’ and ‘Beakpi’ showed no pigmentation in root skin or flesh and were designated as W1 and W2, respectively, whereas the two green root radishes, ‘847’ and ‘IT250777’ showed the typical green color in both root skin and flesh and were named G1 and G2, respectively. We extracted and quantified the chlorophyll contents of root skin and flesh (Figure 2B). In agreement with the visible phenotypes, chlorophyll was barely detectable in the white radishes W1 and W2, whereas the root skin and flesh of G1 and G2 radishes showed a high level of chlorophyll. Interestingly, chlorophyll contents in the skin of green radishes were higher than those in flesh. As expected from the visible skin phenotype, the chlorophyll content in the G1 radish was higher than in the G2 radish, indicating that the amount of chlorophyll accumulation is responsible for the degree of coloration that causes the green pigmentation phenotype.

### 2.2. RsPORB Is Highly Expressed in Green Radish Roots

**Figure 3:**
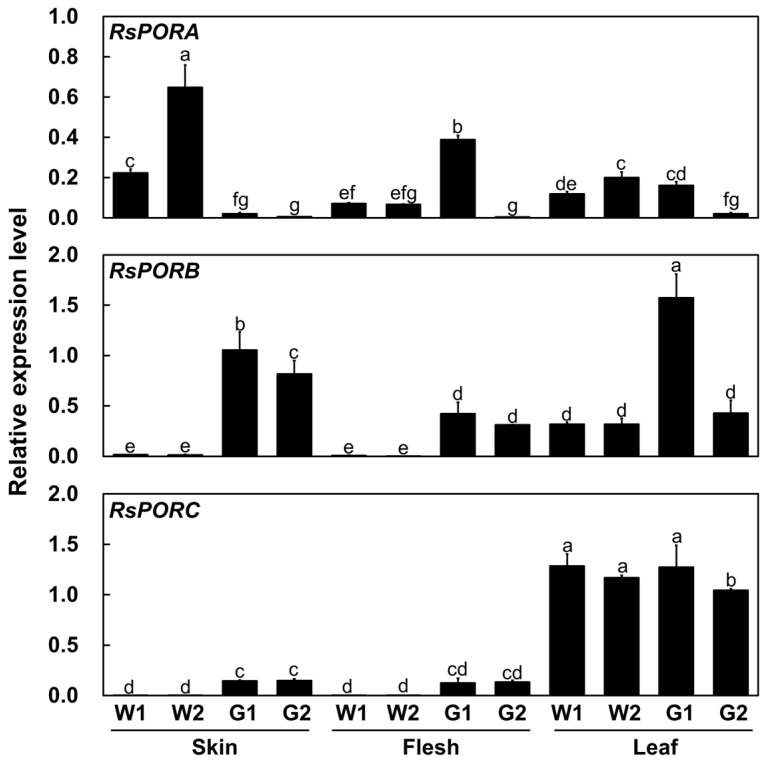
Transcript levels of *RsPORA*, *RsPORB*, and *RsPORC* in root skin, root flesh, and leaf from white (W1, W2) and green (G1, G2) radishes. Results represent the mean values ± SD from three independent biological replicates. Different letters indicate significantly different values (*p* < 0.05), as determined using a one-way ANOVA followed by Duncan’s multiple range tests.

PORs, key enzymes that catalyze the conversion of Pchlide to Chlide, are essential for chlorophyll biosynthesis during plant growth and development. The preliminary test conducted in our group indicated that transcriptomic analysis with the skin of white and green radishes showed different transcript levels of chlorophyll biosynthetic and degradation genes. This result was further supported by several studies that showed the significant transcript level variations of *POR* genes in chlorophyll accumulating tissues from several crops [14,15,16,17]. To validate the relationship between the expression of PORs and chlorophyll content, we analyzed the transcript level of three *POR* genes including *RsPORA* (accession XP_018482038.1), *RsPORB* (accession XP_018468981), and *RsPORC* (accession XP_018477724.1) in the skin and flesh of root and in leaf at mature stages (Figure 3).

In the root skin, the transcript level of *RsPORA* was higher in the white radishes than in the green radishes. Although *RsPORB* and *RsPORC* were highly expressed in the green radishes, they were barely expressed in white radishes. In root flesh, the transcript level of *RsPORA* in the white radishes was lower than that in the G1 radish, but higher than the G2 radish. Similar to the expression pattern in root skin, *RsPORB* and *RsPORC* were highly expressed in all green radishes, but not in the white radishes. In the leaf, *RsPORA* was expressed at a similar, low level in white and green radishes. Whereas the level of *RsPORB* was lower in white radishes than in green radishes, the transcript level of *RsPORC* was high in both white and green radishes. These results indicated that the *RsPORB* transcript pattern correlated well with the chlorophyll contents in the root skin and flesh, suggesting that *RsPORB* is responsible for chlorophyll biosynthesis in non-foliar tissue such as root skin and flesh.

### 2.3. RsPORB Gene Has Highly Conserved Motifs for Chlorophyll Biosynthesis

To elucidate a molecular basis for the phenotypic variation due to differences in chlorophyll biosynthesis, we isolated and compared the nucleotide sequences of the *RsPORB* gene from the G1 and W1 radishes by PCR using primers designed from the previously deposited sequence of the *RsPORB* gene. The coding region sequence of the *RsPORB* genes from both radishes was 100% identical with the previously reported sequences. It was 1197 bp in length and encoded a predicted protein of 398 amino acid residues.

**Figure 4:**
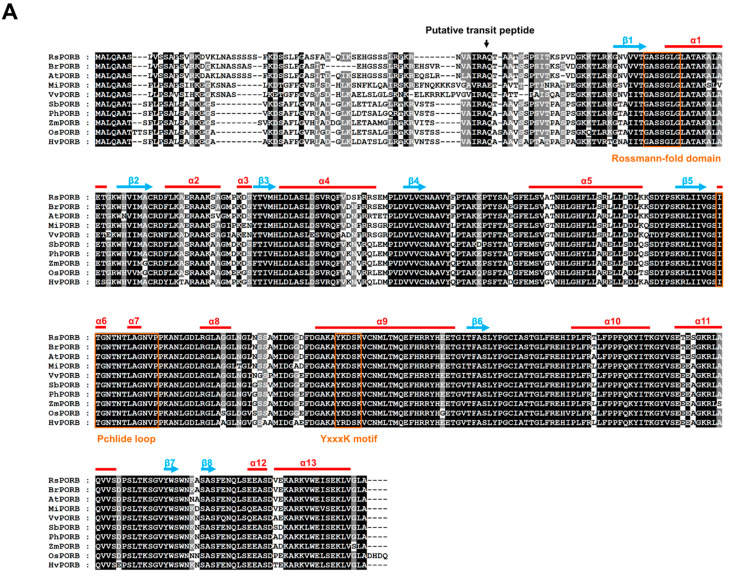

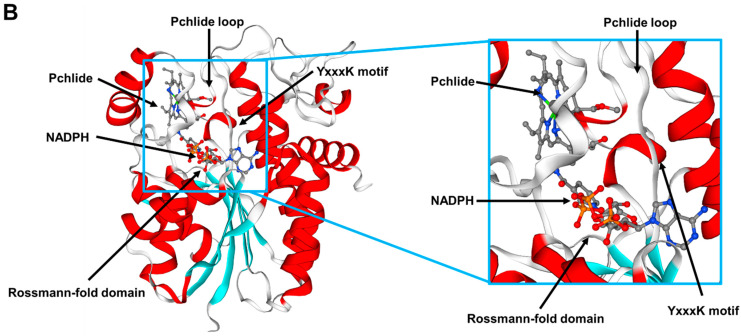
Multiple sequence alignment and *RsPORB* protein structure analysis. (**A**) Multiple sequence alignment of PORB proteins. α-Helices and β-strands are indicated by red and cyan lines, respectively, and the conserved Rossmann-fold domain, Pchlide loop, and YxxxK motif are outlined by orange boxes. The putative N-terminal cleavage sites for the plastid transit peptides are indicated by the arrow. (**B**) Structural modeling of *RsPORB* protein. The 3D protein structure modeling based on PDB ID: 7JK9 from *Arabidopsis thaliana* built using SWISS-MODEL. The α-helices and β-strands are shown in red and cyan, respectively. The enlarged view showed the binding of *RsPORB* protein to Pchlide and NADPH.

Sequence alignment with PORB homologs of other plants showed that *RsPORB* protein displayed highly conserved characteristics (Figure 4A). The putative chloroplast transit peptide at the N terminus that would be cleaved off after targeting to produce the mature protein shows relatively low sequence similarity among PORB proteins, as expected for a transit peptide [18]. PORB proteins belong to the short-chain dehydrogenase/reductase (SDR) superfamily, a group of NAD(P)H-dependent enzymes [19]. SDR proteins are characterized by several conserved motifs, which are either directly involved in NAD(P)H cofactor binding or cluster around the substrate-binding pocket [18]. The common structural features include the Rossmann-fold domain (GxxxGxG) sequence for NADPH binding, a YxxxK motif, and tyrosine and lysine residues at the enzyme catalytic pocket. PORB binds NADPH as a hydrogen donor for substrate reduction through a Rossmann-fold domain located at the N terminus and can participate in establishing the enzyme catalytic pocket in concert with the YxxxK motif (Figure 4B) [19,20]. The Pchlide loop and helix α10 in *RsPORB* are involved in substrate binding [21], which induces a conformational change in helix α10, resulting in POR oligomerization [22,23]. Phylogenetic analysis with *RsPORB* and other known PORB proteins from various plant species indicated that *RsPORB* fell within the well-supported eudicot clade that includes PORBs from *Brassica rapa* and *Arabidopsis*, consistent with their close taxonomic relationship in the Brassicaceae (Supplementary Figure S1). The high conservative characteristics of POR proteins, including NADPH and Pchlide binding sites, assumes that *RsPORB* has the function of chlorophyll biosynthesis in radish.

### 2.4. RsPORB Is Involved in Chlorophyll Biosynthesis

**Figure 5:**
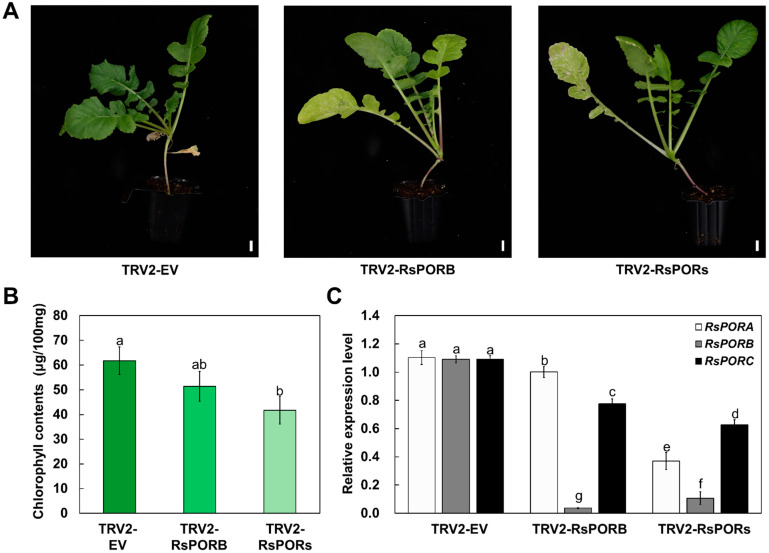
Silencing of *RsPORB* reduces the chlorophyll contents. (**A**) Representative radish plants 3 weeks after post-infiltration with the empty vector as the control (TRV2-EV), *RsPORB* (TRV2-PORB) and *RsPOR* genes (TRV2-PORs). Bar = 1 cm. (**B**) Chlorophyll contents in *RsPORB-* and *RsPOR*-silenced radish leaves. (**C**) Transcript levels of *RsPORA*, *RsPORB*, and *RsPORC* in leaves. Different letters indicate significantly different values (*p* < 0.05), as determined using a one-way ANOVA followed by Duncan’s multiple range tests.

To demonstrate whether *RsPORB* is essential for chlorophyll biosynthesis, we conducted transient expression analyses using virus-induced gene silencing (VIGS) [24]. We introduced either a specific 675 bp fragment of *RsPORB* or a 517 bp fragment that is conserved among *RsPOR* genes into *pTRV2* to generate *pTRV2-RsPORB* or *pTRV2-RsPORs*, respectively, for *in planta* gene silencing. The vector-only *pTRV2-empty* plasmid was used as a negative control. We co-infiltrated *Agrobacterium* strains individually carrying *TRV2-empty*, *TRV2-*RsPORB**, or *TRV2-RsPOR* genes along with *TRV1* into young radish leaves. After 3 weeks, a pale coloration was detected in the leaves following silencing of *RsPORB* and *RsPOR* genes, but no such color appeared in the leaves agroinfiltrated with the empty vector (Figure 5A). Similar to the visible phenotype, the chlorophyll contents were lower in the leaves infiltrated with *pTRV2-RsPORB* and *pTRV2-RsPORs* than in those infiltrated with *pTRV2-empty* (Figure 5B). Accumulation of *RsPORB* and *RsPORs* transcripts was strongly downregulated in the leaves, indicating that these genes were silenced (Figure 5C). Taken together, these results confirmed that *RsPORB* plays an essential role in chlorophyll biosynthesis in radish.

### 2.5. Promoter Region of RsPORB between Green and White Radish Was Variable

**Figure 6:**
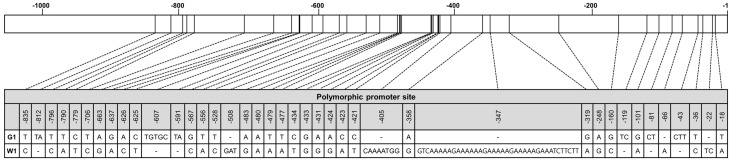
Polymorphic sites in the *RsPORB* promoter region in G1 vs. W1 radishes. Numbers indicate positions from the ATG start codon of *RsPORB* in the G1 radish.

Despite the 100% identity of *RsPORB* coding sequences from G1 and W1 radishes, the transcript level of *RsPORB* in the root skin and flesh was notably higher in green radishes than in white radishes (Figure 3), suggesting that promotor variation might be involved in its expression level. To determine whether the *RsPORB* promoter polymorphisms contributed to the differential pattern of chlorophyll accumulation, we isolated the putative promoter regions of *RsPORB* from G1 and W1 radishes by PCR and designated them as *proRsPORB-G1* (1000 bp) and *proRsPORB-W1* (1038 bp), respectively. A Comparison of the promoter regions of *RsPORB-G1* and *RsPORB-W1* found the presence of 29 single-nucleotide polymorphisms (SNPs) and 11 InDels (Figure 6). Variation in the number of InDels resulted in the different lengths of the *RsPORB* promoters from green vs. white radish.

Several significant putative *cis*-acting elements were identified in *proRsPORB-G1* and *proRsPORB-W1* (Supplementary Figure S2). LONG HYPOCOTYL 5 (HY5), a basic leucine zipper (bZIP) protein, is a positive regulator of chlorophyll biosynthesis and can bind to the CCAAT motif, resulting in activation of photomorphogenesis-related genes [25]. Three and two CCAAT motifs were detected in *proRsPORB-G1* and *proRsPORB-W1*, respectively (Table 1). DNA binding with one finger (Dof) proteins are plant-specific transcription factors that have a dual function in transcriptional control as an activator or a repressor to bind to Dof binding sites (AAAG sequences) for regulating light responsiveness [26,27]. Recent studies reported that the rice (*Oryza sativa*) stay-green (OsSGR) encoding a chlorophyll-degrading Mg^++^-dechelatase exhibited a lower expression level in *japonica*-type rice than in *indica*-type rice. It demonstrated that additional Dof motifs in the *OsSGR* promoter from *indica*-type rice resulted in rapid senescence phenotypes [28]. Additionally, it reported that tea (*Camellia sinensis*) CsCLH1 encoding a chlorophyllase that catalyzes the hydrolysis of phytol from chlorophyll was highly expressed under high light, resulting in the decrease in chlorophyll content in tea leaves [29]. Under shade conditions, the DOF protein CsDOF3 bound to the Dof motifs in the *CsCLH1* promoter and repressed the *CsCLH1* expression, resulting in chlorophyll accumulation. In this study, we identified three and six Dof binding motifs in *proRsPORB-G1* and *proRsPORB-W1*, respectively. Specifically, the 38-nucleotides insertion at position −352 in *proRsPORB-W1* resulted in four more Dof binding sites. Previous studies revealed that related to ABI13/VP1 (RAV1) was induced in leaf maturation and senescence stages and worked as a positive regulator for leaf senescence [30]. We confirmed the number of CAACA motifs targeted by RAV1 to be one in *proRsPORB-G1* and three in *proRsPORB-W1*. Interestingly, additional CAACA motifs and Dof binding sites were created due to the insertions at positions −440 and −352 in *proRsPORB-W1*, respectively. It suggested that sequence variation in *RsPORB* promoters may affect promoter activity and chlorophyll biosynthesis.

### 2.6. Variation of RsPORB Promoter Was Associated with Its Expression Level

**Figure 7:**
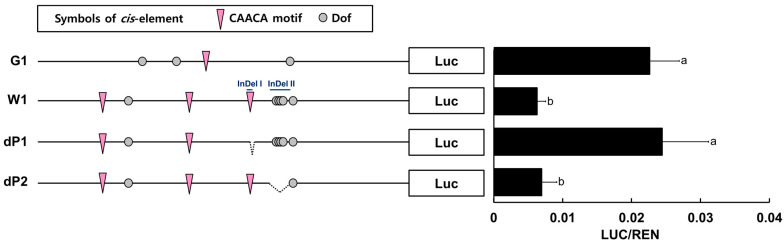
Promoter variation of the *RsPORB* gene can alter its expression level. Schematic representations of *RsPORB* promoter constructs from G1, W1, and constructs with deleted InDel regions of the *RsPORB-W1* promoter (dP1) and dP2 (left). dP1 and dP2 were deleted from −441 to −448 and from −353 to −390 of *RsPORB-W1* promoter, respectively. We used a dual-luciferase promoter plasmid encoding the *firefly luciferase* gene driven by the *RsPORB* promoters G1, W1, dP1, and dP2 as well as a *Renilla luciferase* (*REN*) gene driven by the *CaMV 35S* promoter to measure LUC and REN levels (right). Data denote protoplast accumulation of each fusion protein at 16 h after transfection. Different letters indicate significantly different values (*p* < 0.05), as determined using a one-way ANOVA followed by Duncan’s multiple range tests.

To further investigate the relationship between promoter variation and chlorophyll biosynthesis, we performed promoter activation assays with *proRsPORB-G1* and *proRsPORB-W1* using radish root protoplasts (Figure 7). Transient assays revealed that the promoter activity of *proRsPORB-G1* was 3.6-fold higher than that of *proRsPORB-W1*. This result suggests that variation in the *RsPORB* promoter contributes to the altered expression level of *RsPORB* in radish root.

Through the *cis* element analysis and alignment between the two *RsPORB* promoters, we noticed a distinctive difference in two InDel sites of *proRsPORB-W1* that included different *cis* element sites, such as the CAACA motif and Dof elements. To further define which InDel sites of *proRsPORB-W1* were responsible for variations in its promoter activity, we constructed deletion variants (dP1 and dP2) for each InDel. Transient assays with radish root protoplasts revealed that promoter activity derived from the dP1 construct can recover to the level seen with the *RsPORB-G1* promoter, suggesting that the CAACA motif is to be a crucial target for putative negative regulators such as RAV1. Meanwhile, the promoter activity due to the dP2 construct was similar to that of *RsPORB-W1*, indicating that eliminating the Dof motif had a negligible effect on promoter activation. These results suggest that the differential expression level of *RsPORB* in radish roots is due to variations in its promoter.

## 3. Discussion

We have shown here that *RsPORB,* one of the key genes for chlorophyll biosynthesis, showed a differential expression level in radish roots due to its promoter variation. Defining the chlorophyll biosynthetic mechanism in non-foliar tissues has significant implications for the development of novel plant and fruit varieties with various chlorophyll accumulation patterns, through either conventional or advanced breeding methods.

### 3.1. Expression of RsPORB Activates Chlorophyll Biosynthesis in Radish Roots

POR is a key enzyme required for chlorophyll biosynthesis that catalyzes the reduction of Pchlide to Chlide [4,5,7]. Here, we confirmed that three *RsPORs* including *RsPORA*, *RsPORB* and *RsPORC* exhibited the different expression profiles (Figure 3). The expression levels of *RsPORB* and *RsPORC* in chlorophyll-accumulating tissues, including root skin, root flesh, and leaf, were high, but the *RsPORA* transcript level was relatively low. High transcript levels of *RsPORB* were detected in all of the root skin and root flesh from green radishes, implying that *RsPORB* has a crucial role in chlorophyll biosynthesis in non-foliar tissues. Additionally, *RsPORC* was strongly expressed in mature leaf, suggesting that *RsPORC* is crucial for chlorophyll biosynthesis and maintenance in foliar tissue. Similarly, a previous study reported that the expression level of *RsPORB* and *RsPORC* was higher in green-fleshed radish had compared to white-fleshed radish [14]. Similarly, the distinct physiological roles of three POR isoforms in chlorophyll biosynthesis have been identified in *Arabidopsis* [8,9,31]. *AtPORA* is highly expressed in etiolated seedlings and emerging cotyledons, and then expression is rapidly diminished by illumination. The *AtPORB* transcript is detected not only in both etiolated seedlings and light-grown seedlings, but also in chlorophyll-containing organs of adult plants. Expression of *AtPORC* is very low during skotomorphogenesis but elevated during greening. The expression of *AtPORB* and *AtPORC* is regulated by circadian oscillation and the fluence rate, respectively, and is abundant in greening tissues. Arabidopsis plants with individual mutations in *atporB* and *atporC* exhibit normal photosynthetic competencies, suggesting that AtPORB and AtPORC are interchangeable and functionally redundant in the maintenance of light-dependent chlorophyll biosynthesis during plant development [32].

Furthermore, VIGS assays verified that the expression of *RsPORB* and *RsPOR* genes is crucial for chlorophyll biosynthesis (Figure 5). The silencing of *RsPORB* and *RsPOR* genes led to a decrease in chlorophyll content, resulting in pale coloration. These results confirmed that both *RsPORB* and RsPORs are not only functional enzymes for chlorophyll biosynthesis but also differential expression patterns in both foliar and non-foliar tissues, resulting in differential green colors depending on the tissues.

### 3.2. Promoter Variation of RsPORB Affects Its Transcript Level

Transcriptional factors (TFs) can activate or repress target gene expression via binding to *cis* elements within promoter regions. Therefore, promoter variation can spatially and temporally alter the transcript level of its gene. Here, in checking how promoter variation affects the expression level of the *RsPORB*, we observed many polymorphisms, including SNPs and InDels, in the promoter region of *RsPORB-W1* compared with those of *RsPORB-G1* (Figure 6). Among them, the additional CAACA motif and Dof elements were presented in the promoter region of *RsPORB-W1* by the insertion of InDels. Interestingly, the deletion (dP1) of proximal *cis* elements out of three CAACA motifs in *proRsPORB-W1* elevated the level of promoter activation and can be restored to the level of *RsPORB-G1* promoter activation, despite the presence of additional two CAACA motifs (Figure 7), whereas deleted Dof elements (dP2) in *proRsPORB-W1* showed the same promoter activity level of native *proRsPORB-W1*, implying that the additional Dof elements in *proRsPORB-W1* were insignificantly affected in its expression level. These results indicated that variation of *cis* elements in the *RsPORB* promoter can change its own expression level, underlying the chlorophyll contents.

Similarly, the promoter variation of chlorophyll-related genes showed the phenotypic variation due to the differential chlorophyll biosynthesis and degradation. The T-DNA insertion in the promoter of *Arabidopsis* AtCHL27, which encodes a catalytic subunit of Mg-protoporphyrin monomethyl ester cyclase, exhibited growth retardation, chloroplast developmental defects, and chlorophyll content reduction [33]. As well, promoter variation in *OsSGR* causes different chlorophyll accumulation and senescence patterns in rice leaf [28]. The *indica*-type rice cultivars usually show relatively faster senescence and chlorophyll loss compared with *japonica*-type rice cultivars, due to variation in the *OsSGR* promoters. These variations are expected to involve the additional insertion of the Dof-binding motif in its promoter region, resulting in the acceleration of chlorophyll loss. Several studies have reported that the HY5 TF, a pivotal factor regulating photomorphogenesis and chloroplast development, can specifically bind to the promoters of light-responsive genes via the G-box element and CCAAT motif [34]. Indeed, deletion of the G-box sequence in the promoter of the *AtCHLH* gene encoding the H subunit of Mg-chelatase resulted in the absence of its expression in roots but not in shoots, highlighting how promoter variation can influence organ-specific expression [35].

Several studies reported that interaction between transcription factors elaborately regulates the chlorophyll biosynthetic genes in a spatial and temporal manner in different tissues [36,37]. HY5, a key transcription factor for light signaling, binds to the common cis element (G box) in *CHLH* and *PORC* promoter and activates its expression level, resulting in chlorophyll accumulation [36]. Recently, it was reported that the CINCINNATA-like TEOSINTE BRANCHED 1/CYCLOIDEA/PCF (TCP) proteins act as repressors for chlorophyll accumulation [37]. Arabidopsis plant with septuple *tcp2/3/4/5/10/13/17* mutant has the green petals coloration via chlorophyll accumulation. Additionally, the AtTCP4 protein directly binds to promoters of *AtPORB* and *AtDVR* and represses their expression, resulting in preventing chlorophyll accumulation.

Taken together, these results suggest that the precise genome editing of regulatory genes or *cis* elements in the PORs promoter could be an effective strategy for developing crops with desired coloration patterns.

## 4. Materials and Methods

### 4.1. Plant Materials

Different root-colored radishes (*Raphanus sativus*) were used in this study. Two white radishes have white skin and flesh: W1, ‘948’ (Asia Seed Co., Seoul, Republic of Korea), and W2, ‘Beakpi’ (Nongwoo Seed Co., Suwon, Republic of Korea). Two green radishes have green skin and flesh: G1, ‘847’ (Asia Seed Co.), and G2, ‘IT250777’ (National Agrobiodiversity Center at the National Institute of Agricultural Sciences, Jeonju, Republic of Korea). Radish plants were grown for 8 weeks from sowing in the field in autumn at the National Institute of Agricultural Sciences.

### 4.2. Measurement of Chlorophyll Contents

To analyze chlorophyll contents, root skin and flesh were rapidly frozen in liquid nitrogen and stored at −80 °C. Each sample was ground to a fine powder under liquid nitrogen, and chlorophyll contents were measured as previously described [38]. Briefly, aliquots of 100 mg (fresh weight) of powdered tissue samples were incubated in 1 mL 80% acetone for 2 h at 25 °C with rotation at 120 rpm. To precipitate plant debris, samples were centrifuged at 19,000× *g* for 5 min at 4 °C. Absorbance of the supernatant was recorded at 664 nm (*A*_664_) and 647 nm (*A*_647_) using a spectrophotometer (Optizen POP; Mecasys, Daejeon, Republic of Korea), and chlorophyll contents were calculated as follows [39]: chlorophyll *a* = (12.63 × *A*_664_) − (2.52 × *A*_647_); chlorophyll *b* = (20.47 × *A*_647_) − (4.73 × *A*_664_); and chlorophyll contents was the sum of chlorophyll *a* and chlorophyll *b* contents.

### 4.3. Total RNA Extraction and Quantitative Real-Time Polymerase Chain Reaction (qRT-PCR)

Total RNA was extracted from root skins, flesh, and leaves of each of the four radish cultivars using the Fruit-mate for RNA purification solution (Takara, Otsu, Japan) and Plant Total RNA Mini Kit (Farvorgen, Changzhi, Taiwan) as described previously [40]. First-strand cDNA was synthesized from 1 μg of total RNA using the amfiRivert cDNA Synthesis Platinum Master Mix (GenDEPOT, Barker, TX, USA). Transcript levels were measured by qRT-PCR using the AccuPower 2x Greenstar qPCR Master Mix (Bioneer, Daejun, Republic of Korea) and a Bio-Rad CFX96 Detection System (Bio-Rad Laboratories, Hercules, CA, USA) according to the manufacturer’s instructions. The expression levels of all target genes were normalized to that of the radish *RNA polymerase II* (*RsRPII*) gene (accession XP_018434834) as an internal reference. Three independent biological samples served as replicates. The gene-specific primers used for RT-qPCR analysis are listed in Supplementary Table S1.

### 4.4. Sequence Analysis of Promoter and Coding DNA Sequences of RsPORB

Genomic DNA was extracted using a Genomic DNA Prep Kit (Inclone, Yongin, Republic of Korea). The coding and promoter regions of *RsPORB* were amplified from cDNA and genomic DNA of G1 and W1 from radish root skins and leaves, respectively, with PrimeSTAR HS DNA Polymerase (Takara) using the primer pairs *RsPORB-F*/*R* and *RsPORB-promoter-F*/*R*, respectively (Supplementary Table S1). All amplified PCR products were subcloned into the pTOP Blunt V2 vector (Enzynomics, Daejeon, Republic of Korea) for validation by sequencing.

Multiple sequence alignments were generated using CLUSTALW (https://www.genome.jp/tools-bin/clustalw, accessed on 7 April 2023). A phylogenetic tree was constructed with MEGAX software using the neighbor-joining method [41]. The GenBank accession numbers and species used in this study are as follows: AtPORB, *Arabidopsis thaliana*, NP_001031731.1; BrPORB, *Brassica rapa*, XP_009143453; HvPORB, *Hordeum vulgare*, XP_044968468; MiPORB, *Macadamia integrifolia*, XP_042495636; OsPORB, *Oryza sativa*, XP_015614945.1; PhPORB, *Panicum hallii*, XP_025796366; SbPORB, *Sorghum bicolor*, XP_002467010; VvPORB, *Vitis vinifera*, RVX09506; ZmPORB, *Zea mays*, PWZ55469.1. The 3D structure of the *RsPORB* protein was built using the SWISS-MODEL server (https://swissmodel.expasy.org, accessed on 7 April 2023) [42]. The homology model was generated using *Arabidopsis thaliana* PORB (PDB ID: 7JK9) as a template.

### 4.5. Construction of RsPORB and RsPOR VIGS Vectors and Agrobacterium-Mediated Infiltration

For VIGS analysis, the 675 bp fragment from the *RsPORB*-specific region and the 517 bp fragment from the conserved region of *RsPOR* genes were separately cloned into *pTRV2-LIC* [22]. The primers used for amplification are listed in Supplementary Table S1. The vectors *pTRV2-RsPORB*, *pTRV2-RsPORs*, *pTRV2-empty*, and *pTRV1* were each transformed into the *Agrobacterium* strain GV3101. Transformed GV3101 lines were cultured at 28 °C with YEP medium and resuspended to an OD_600_ of 0.8 in infiltration buffer containing 10 mM MgCl_2_, 10 mM MES (pH 5.7), and 150 μM acetosyringone. Suspensions were incubated at room temperature without shaking for 2 h. *Agrobacteria* harboring *pTRV2* and *pTRV1* constructs were mixed at a ratio of 1:1 (*v/v*). The infiltrated radish plants were kept in the dark overnight and then grown under LD conditions. After 21 days, the radish leaves were photographed and collected for further analysis.

### 4.6. Promoter Activation Assay in Radish Root Protoplasts

The *RsPORB* promoter regions from radish cultivars G1 and W1 were individually amplified by PCR and cloned into the *pUC-fLUC* vector for reporter constructs [43]. Two additional modified constructs (dP1 and dP2) were generated by the integration of DNA fragments that were amplified by the specific primer sets for the deletion of the promoter region of *RsPORB-W1*. Then, resultant products were subcloned into the *pTOP Blunt V2* vector (Enzynomics) for validation by sequencing. For, the report constructs, full-length PCR products with dP1 and dP2 were individually inserted into *pUC-fLUC* vector digested with *Bam*HI and *Nco*I using an In-Fusion HD Cloning Kit (Takara). *Renilla* luciferase (rLUC) driven by the *UBIQUITIN 10* promoter of *Arabidopsis* was used as an internal control.

For radish root protoplast preparation, seeds of radish cv. Jinjudaepyong were sterilized with surface-sterilized using 70% (*v/v*) ethanol and 0.01% (*v/v*) Triton X-100 before being sown onto half-strength Murashige and Skoog medium [44] (Duchefa, Haarlem, The Netherlands) with 1% sucrose and incubated at 24 °C for 3 weeks after stratification at 4 °C for 2 days. Protoplasts were prepared from radish roots using a slightly modified methods as previously described [40]. Briefly, a bundle of radish roots was chopped and incubated in the protoplast isolation solution (1.5% cellulose R-10, 0.4% macerozyme R-10, 0.4 M mannitol, 20 mM KCl, 20 mM MES at pH5.7, 0.1% BSA, and 10 mM CaCl_2_) for 4 h in dark with gentle shaking (50 rpm). Isolated root protoplasts were diluted with one volume of W5 solution (125 mM CaCl_2_, 154 mM NaCl, 5 mM KCl, and 2 mM MES at pH 5.6) and filtrated through 40 μm mesh. After brief centrifugation at 100× *g* for 5 min, the collected protoplasts were resuspended in W5 solution, and incubated on ice for 30 min. Then, root protoplasts were collected by brief centrifugation and resuspended in MMG solution (0.4 M mannitol, 15 mM MgCl_2_, and 4 mM MES at pH 5.6). The root protoplasts (1 × 10^6^ protoplasts) were transformed by PEG-mediated methods with 10 μg plasmid DNA at 22 °C for 30 min incubation. After stopping the transfection by adding W5 solution, collected protoplasts were resuspended with W5 solution and incubated at 22 °C for 16 h. The firefly luciferase (LUC) and *Renilla* luciferase (REN) levels were measured using a dual luciferase assay system (Promega, Madison, WI, USA) according to the manufacturer’s protocol. Normalized reporter activity was calculated from the LUC/REN ratio. The primers used for the constructs in promoter activation analysis are listed in Supplementary Table S1.

## 5. Conclusions

We demonstrated that *RsPORB* is a key gene stimulating chlorophyll biosynthesis in radish roots. The expression of *RsPORB* was positively correlated with chlorophyll content in root skin and root flesh of green radishes. Sequence analysis revealed that the coding region sequences of *RsPORB* were identical between white and green radish, but the promoter regions showed significant differences. Additionally, silencing of the *RsPORB* and *RsPOR* genes confirmed that they play crucial roles in chlorophyll biosynthesis. Interestingly, a considerable number of nucleotide variations, such as InDels and SNPs, were detected in promoter regions of *RsPORB* in white vs. green radishes. Promoter activation assays with radish root protoplasts revealed that nucleotide variation in the *RsPORB* promoter can contribute to its expression level. Taken together, these results indicated that the expression level of *RsPORB* plays a key role in chlorophyll biosynthesis in radish roots.

## Figures and Tables

**Table 1:** Putative *cis*-acting elements in the *RsPORB* promoter from the G1 and W1 radishes.

Motif	Sequence	Position (from ATG codon)		Recognized by
		G1	W1	
CCAAT	CCAAT	−835 to −839, −310 to −314, −273 to −277	−308 to −312, −271 to −275	HY5
Dof	AAAG	−757 to −760, −660 to −663, −333 to −336	−797 to −800, −374 to −377, −367 to −370, −361 to −364, −355 to −358, −331 to −334	Dof
CAACA	CAACA	−575 to −579	−872 to −876, −622 to −626, −447 to −451	RAV1

## Data Availability

Data supporting the findings of this study are available from the corresponding author, Sun-Hyung Lim, upon request.

## Supplementary Materials

The following supporting information can be downloaded at: https://www.mdpi.com/article/10.3390/plants12112214/s1, Table S1: Gene-specific primers used in this study. Figure S1: Phylogenetic relationships among PORB proteins from radish and other plants. Figure S2: Nucleotide sequences of *RsPORB* promoters from radish cultivars G1 and W1.

## References

[1] Tripathy B.C., Oelmüller R. (2012). Reactive oxygen species generation and signaling in plants. Plant Signal. Behav..

[2] Pospíšil P. (2016). Production of reactive oxygen species by photosystem II as a response to light and temperature stress. Front. Plant Sci..

[3] Fu H., Zeng T., Zhao Y., Luo T., Deng H., Meng C., Luo J., Wang C. (2021). Identification of chlorophyll metabolism-and photosynthesis-related genes regulating green flower color in chrysanthemum by integrative transcriptome and weighted correlation network analyses. Genes.

[4] Masuda T., Takamiya K. (2004). Novel insig hts into the enzymology, regulation and physiological functions of light-dependent protochlorophyllide oxidoreductase in angiosperms. Photosynth. Res..

[5] Scrutton N.S., Groot M.L., Heyes D.J. (2012). Excited state dynamics and catalytic mechanism of the light-driven enzyme protochlorophyllide oxidoreductase. Phys. Chem. Chem. Phys..

[6] Reinbothe C., El Bakkouri M., Buhr F., Muraki N., Nomata J., Kurisu G., Fijita Y., Reinbothe S. (2010). Chlorophyll biosynthesis: Spotlight on protochlorophyllide reduction. Trends Plant Sci..

[7] Reinbothe S., Reinbothe C., Holtorf H., Apel K. (1995). Two NADPH: Protochlorophyllide oxidoreductases in barley: Evidence for the selective disappearance of PORA during the light-induced greening of etiolated seedlings. Plant Cell.

[8] Masuda T., Fusada N., Oosawa N., Takamatsu K., Yamamoto Y.Y., Ohto M., Nakamura K., Goto K., Shinata D., Shirano Y. (2003). Functional analysis of isoforms of NADPH:protochlorophyllide oxidoreductase (POR), PORB and PORC, in *Arabidopsis thaliana*. Plant Cell Physiol..

[9] Paddock T.N., Mason M.E., Lima D.F., Armstrong G.A. (2010). Arabidopsis protochlorophyllide oxidoreductase A (PORA) restores bulk chlorophyll synthesis and normal development to a *porB porC* double mutant. Plant Mol. Biol..

[10] Holtorf H., Reinbothe S., Reinbothe C., Bereza B., Apel K. (1995). Two routes of chlorophyllide synthesis that are differentially regulated by light in barley (*Hordeum vulgare* L.). Proc. Natl. Acad. Sci. USA.

[11] Gutiérrez R.M.P., Perez R.L. (2004). *Raphanus sativus* (Radish): Their chemistry and biology. Sci. World J..

[12] Park N.I., Xu H., Li X., Jang I.H., Park S., Ahn G.H., Lim Y.P., Kim S.J., Park S.U. (2011). Anthocyanin accumulation and expression of anthocyanin biosynthetic genes in radish (*Raphanus sativus*). J. Agric. Food Chem..

[13] Park C.H., Ki W., Kim N.S., Park S.Y., Kim J.K., Park S.U. (2022). Metabolic Profiling of White and Green Radish Cultivars (*Raphanus sativus*). Horticulturae.

[14] Li Y.Y., Han M., Wang R.H., Gao M.G. (2021). Comparative transcriptome analysis identifies genes associated with chlorophyll levels and reveals photosynthesis in green flesh of radish root. PLoS ONE.

[15] Liu T., Zhang Y., Zhang X., Sun Y., Wang H., Song J., Li X. (2019). Transcriptome analyses reveal key genes involved in skin color changes of ‘Xinlimei’radish root. Plant Physiol. Biochem..

[16] Ohmiya A., Sasaki K., Nashima K., Oda-Yamamizo C., Hirashima M., Sumitomo K. (2017). Transcriptome analysis in petals and leaves of chrysanthemums with different chlorophyll levels. BMC Plant Biol..

[17] Kwon C.T., Kim S.H., Song G., Kim D., Paek N.C. (2017). Two NADPH: Protochlorophyllide Oxidoreductase (POR) Isoforms Play Distinct Roles in Environmental Adaptation in Rice. Rice.

[18] Gabruk M., Stecka A., Strzałka W., Kruk J., Strzałka K., Mysliwa-Kurdziel B. (2015). Photoactive protochlorophyllide-enzyme complexes reconstituted with PORA, PORB and PORC proteins of *A. thaliana*: Fluorescence and catalytic properties. PLoS ONE.

[19] Bray J.E., Marsden B.D., Oppermann U. (2009). The human short-chain dehydrogenase/reductase (SDR) superfamily: A bioinformatics summary. Chem. Biol. Interact..

[20] Buhr F., El Bakkouri M., Valdez O., Pollmann S., Lebedev N., Reinbothe S., Reinbothe C. (2008). Photoprotective role of NADPH: Protochlorophyllide oxidoreductase A. Proc. Natl. Acad. Sci. USA.

[21] Nguyen H.C., Melo A.A., Kruk J., Frost A., Gabruk M. (2021). Photocatalytic LPOR forms helical lattices that shape membranes for chlorophyll synthesis. Nat. Plants.

[22] Zhang S., Godwin A.R.F., Taylor S., Hardman S.J.O., Jowitt T.A., Johannissen L.O., Hay S., Baldock C., Heyes D.J., Scrutton N.S. (2021). Dual role of the active site ‘lid’ regions of protochlorophyllide oxidoreductase in photocatalysis and plant development. FEBS J..

[23] Menon B.R., Hardman S.J., Scrutton N.S., Heyes D.J. (2016). Multiple active site residues are important for photochemical efficiency in the light-activated enzyme protochlorophyllide oxidoreductase (POR). J. Photochem. Photobiol.

[24] Liu Y., Schiff M., Dinesh-Kumar S.P. (2002). Virus-induced gene silencing in tomato. Plant J..

[25] Nawkar G.M., Kang C.H., Maibam P., Park J.H., Jung Y.J., Chae H.B., Chi Y.H., Jung I.J., Kim W.Y., Yun D.J. (2017). HY5, a positive regulator of light signaling, negatively controls the unfolded protein response in *Arabidopsis*. Proc. Natl. Acad. Sci. USA.

[26] Ruta V., Longo C., Lepri A., De Angelis V., Occhigrossi S., Costantino P., Vittorioso P. (2020). The DOF transcription factors in seed and seedling development. Plants.

[27] Kang W.H., Kim S., Lee H.A., Choi D., Yeom S.I. (2016). Genome-wide analysis of Dof transcription factors reveals functional characteristics during development and response to biotic stresses in pepper. Sci. Rep..

[28] Shin D., Lee S., Kim T.H., Lee J.H., Park J., Lee J., Lee J.Y., Cho L.H., Choi J.Y., Lee W. (2020). Natural variations at the *Stay-Green* gene promoter control lifespan and yield in rice cultivars. Nat. Commun..

[29] Liu W., Liu S., Zhang K., Xie M., Sun H., Huang X., Zhang L., Li M. (2022). Chlorophyllase is transcriptionally regulated by CsMYB308/CsDOF3 in young leaves of tea plant. Hortic. Plant J..

[30] Woo H.R., Kim J.H., Kim J., Kim J., Lee U., Song I.J., Kim J.H., Lee H.Y., Nam H.G., Lim P.O. (2010). The RAV1 transcription factor positively regulates leaf senescence in *Arabidopsis*. J. Exp. Bot..

[31] Armstrong G.A., Runge S., Frick G., Sperling U., Apel K. (1995). Identification of NADPH:protochlorophyllide oxidoreductases A and B: A branched pathway for light-dependent chlorophyll biosynthesis in *Arabidopsis thaliana*. Plant Physiol..

[32] Frick G., Su Q., Apel K., Armstrong G.A. (2003). An *Arabidopsis porB porC* double mutant lacking light-dependent NADPH: Protochlorophyllide oxidoreductases B and C is highly chlorophyll-deficient and developmentally arrested. Plant J..

[33] Bang W.Y., Jeong I.S., Kim D.W., Im C.H., Ji C., Hwang S.M., Kim S.W., Son Y.S., Jeong J., Shiina T. (2008). Role of *Arabidopsis* CHL27 protein for photosynthesis, chloroplast development and gene expression profiling. Plant Cell Physiol..

[34] Gangappa S.N., Botto J.F. (2016). The multifaceted roles of HY5 in plant growth and development. Mol. Plant.

[35] Kobayashi K., Obayashi T., Masuda T. (2012). Role of the G-box element in regulation of chlorophyll biosynthesis in Arabidopsis roots. Plant Signal. Behav..

[36] Huang Y., Bai X., Cheng N., Xiao J., Li X., Xing Y. (2020). Wide Grain 7 increases grain width by enhancing H3K4me3 enrichment in the OsMADS1 promoter in rice (*Oryza sativa* L.). Plant J..

[37] Wu X., Liang Y., Gao H., Wang J., Zhao Y., Hua L., Yuan Y., Wang A., Zhang X., Liu J. (2021). Enhancing rice grain production by manipulating the naturally evolved cis-regulatory element-containing inverted repeat sequence of OsREM20. Mol. Plant.

[38] Kim D.H., Park S., Lee J.Y., Ha S.H., Lee J.G., Lim S.H. (2018). A rice B-Box protein, OsBBX14, finely regulates anthocyanin biosynthesis in rice. Int. J. Mol. Sci..

[39] Inskeep W.P., Bloom P.R. (1985). Extinction coefficients of chlorophyll a and b in N, N-dimethylformamide and 80% acetone. Plant Physiol..

[40] Kim D.H., Lee J., Rhee J., Lee J.Y., Lim S.H. (2021). Loss of the R2R3 MYB Transcription Factor RsMYB1 Shapes Anthocyanin Biosynthesis and Accumulation in *Raphanus sativus*. Int. J. Mol. Sci..

[41] Kumar S., Stecher G., Li M., Knyaz C., Tamura K. (2018). MEGA X: Molecular evolutionary genetics analysis across computing platforms. Mol. Biol. Evol..

[42] Arnold K., Bordoli L., Kopp J., Schwede T. (2006). The SWISS-MODEL workspace: A web-based environment for protein structure homology modelling. Bioinformatics.

[43] Lim S.H., Kim D.H., Lee J.Y. (2022). RsTTG1, a WD40 Protein, Interacts with the bHLH Transcription Factor RsTT8 to Regulate Anthocyanin and Proanthocyanidin Biosynthesis in *Raphanus sativus*. Int. J. Mol. Sci..

[44] Murashige T., Skoog F. (1962). A revised medium for rapid growth and bio assays with tobacco tissue cultures. Physiol. Plant..

