# Supplementary material for: Expression of RsPORB Is Associated with Radish Root Color
Source: Plants (Basel). 2023 Jun 3;12(11):2214. doi: 10.3390/plants12112214 (PMC10255411; doi:10.3390/plants12112214)
Supplement: Supplementary file 1 [file plants-12-02214-s001.zip › plants-2360981-supplementary.pdf]

Table S1. Gene specific primers used in this study

Usage	Primer name	Primer sequence (5'-3')
Gene cloning	RsPORB-promoter-F	AATGTTCATGGACAATCCTCCTGATTGT
	RsPORB-promoter-R	TTGAAGGGCCATTTTTGGACAAAACAGAGA
	RsPORB-F	ATGGCCCTTCAAGCCGCTTCTTTGGTC
	RsPORB-R	TTAGGCCAAGCCAACCAGCTTCTCAC
Gene expression	RsPORA-F	ACGGTGAAAAGGCATATAAA
	RsPORA-R	GCTCTCTGAACAAACCTGTC
	RsPORB-F	GACAGAGTCTGGCAAAAGAC
	RsPORB-R	TGATCTCCCAAACTTTACG
	RsPORC-F	CAGAGAACACATACCCCTGT
	RsPORC-R	CACCTGTGCTAGTCTTTTCC
	RsRPII-F	ATCACGCTAAATGGTCTCCT
	RsRPII-R	GCTGCTCTCAATCAAGTCAATC
Virus-induced gene silencing	pTRV2-RsPORB-F	CGACGACAAGACCCTAAAGATGTAAAACTGA
	pTRV2-RsPORB-R	GAGGAGAAGAGCCCTAGCTTTCGGTGGTACGT
	pTRV2-RsPORs-F	CGACGACAAGACCCTAAATGGCACGTGAT
	pTRV2-RsPORs-R	GAGGAGAAGAGCCCTACATGTTGCACACTT
Promoter activation assay	RsPORB-promoter-F	AATGTTCATGGACAATCCTCCTGATTGT
	RsPORB-dP1-R	GTTTGGATAATTTTCTTGGTTTATAATTTTTA
	RsPORB-dP1-F	TAAAAATTATAAACCAAGAAAATTATCCAAAC
	RsPORB-dP2-R	CTTTGGCATGGGAGAAGAAGAAGATATCCTACAA
	RsPORB-dP2-F	TTGTAGGATATCTTCTTCTTCTCCCATGCCAAAG
	RsPORB-promoter-R	TTGAAGGGCCATTTTTGGACAAAACAGAGA
	pUC-RsPORB-fLUC-F	AGGCTCTAGAGGATCCAATGTTCATGGACAATCC
	pUC-RsPORB-fLUC-R	TTGGCGTCTTCCATGGTTTTGGACAAAACAGA

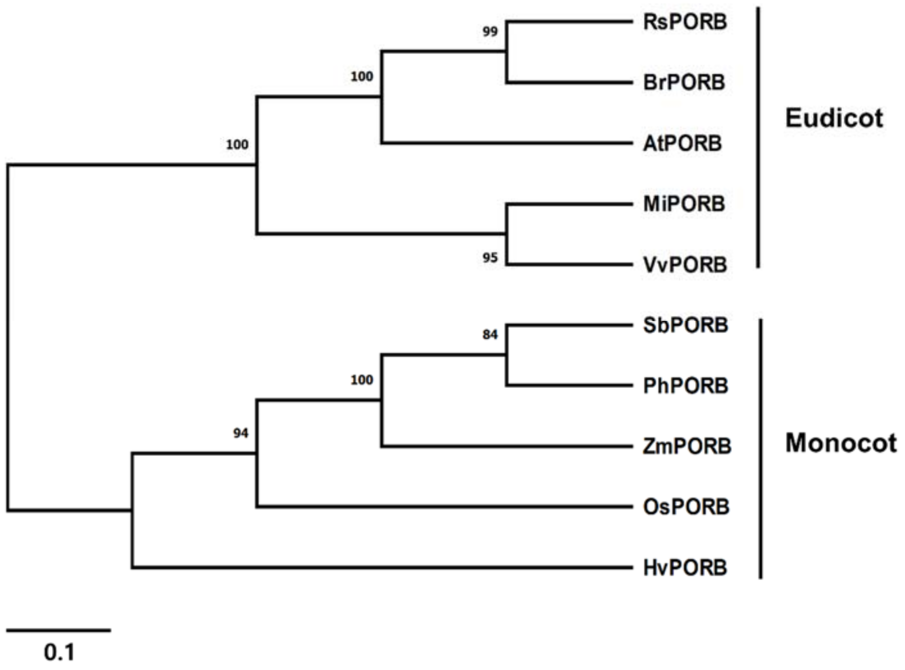

Figure S1. Phylogenetic relationships among PORB proteins from radish and other plants. The phylogenetic tree was constructed using the neighbor-joining method with MEGAX software. Numbers next to the nodes are bootstrap values from 1000 replications.

A

proRsPORB-G1

```
-1000                                  AATGTTCATG GACAATCCTC CTGATTGTCA AACTCTTCGT
 -960 AAGGGTGTCT TCGCTGCGGG TGCCTCCTTT GTCTTCTTCA ACGCCATTGT TTCTCAGTTC
 -900 TATTATTTCT TCTACTCCTC TGCTGCTGCC GCCTCCCTCT CGCCTTACTA GAGGATTTGA
 -840 ACCAATAAAA ATAAGTTTGT TTATTTCTTA TGTGTTATGT GATATCATAC TAATGTCATA
 -780 TCTTGGGCCT GTTGCTTGTT AAAGTATATG ACTGCAAAAT TACTTGAAGT TGAGGGTACG
 -720 ACTATGAGTT TTGTTAGATG GATTTGTATG GATATTGAAT TGAAATGTTG TTCTTAAAAA
 -660 GCTGATCTAA TGTGGTTTTT ACCGAACAAA AAAAAACCAG TGTGCTAGAC TGCTGTGCTA
 -600 TATGTTATAT ATTTGTTAAT TCAACACATG TTAGAAGTTT TATATATGCA ACTAATAGTT
 -540 GTAAGATGAT GGTAGAGCAT GAACAATATG ATAAATAATA TATGTCTTTA TTACCTTAAA
 -480 TTTTGAAAAT TGCTTGTTAA ATTTCAGTTA CACTTTAAAA TTACTTCGAA TTATATACAC
 -420 AAAAATTATA AACCAAGAAA ATTATCCAAA CATATCAGAG TAACCATGAC GAATAGTTTG
 -360 TAAGATATCT TCTTCTCCCA TGCCAAAGTA TGGAATCCCA TGAACACCAA TCACCATCCT
 -300 TTATTGCAAT GTTACCAAAA CAGCCAATAA AATAAAATAT CTACTGAGAG GAAAATCTAG
 -240 CCCTCGAATT CTCATTGGAT ATCTAATGAT GATGTGGCAG ATACTTTCTT TAAGATAATG
 -180 TTATCCACAC ATCTTCTCTG TGATTTCTCT GGGGACCACT CTCTCCCTTG TCCGAACTTC
 -120 TCACCTCCTC CACTCCTTCG CGAGTATACA TAAACGCGTC TACACTCTCG CAAAATATCA
  -60 ACTTCAGTAG TCTTCTTCTT CATTGCTATT GCTATCTCTC ACTCTCTGTT TTGTCCAAAA
```

CCAAT motif Dof CAACA motif

B

proRsPORB-W1

```
-1038                                                        AATGTTCA TGGACAATCC
-1020 TCCTGATTGT CAAACTCTTC GTAAGGGTGT CTTCGCTGCG GGTGCCTCCT TTGTCTTCTT
 -960 CAACGCCATT GTTTCTCAGT TCTATTATTT CTTCTACTCC TCTGCTGCTG CCGCCTCCCT
 -900 CTCGCCTTAC TAGAGGATTT GAACCAACAA AAATAAGTTT GTTTATTTCT TGTGTTATGT
 -840 GATACCATAC AAATGTCATA TTTTGGGCCT GTTGCTTGTT AAAGTATATG ACTGCAAAAT
 -780 TACTTGAAGT TGAGGGTACG ACTATGAGTT TTGTCAGATG GATTTGTATG GATATTGAAT
 -720 TGAAATGTTG TTCTTAAGAA GCTGATCTAA TGTGGTTTTT ACCAAACAAA AAAAACTCAG
 -660 TGTGCTAGAC TGCTATATGT TATATTTGTT AATTCAACAC ATGTTACAAG TTTTATAAAT
 -600 GCAACTAATA GTTGTAAGAT GATGGCAGAG CATGAACAAT ATGATAGATA ATAATATATG
 -540 TCTTTATTAC CTTGAAAATA GAAAATTGCT TGTTAAATTT CAGTTACACT TTAAAATTAC
 -480 TTTGAGTTAT ATGAATAAAA ATTATAAACC AACAAAATGG GAAAATTATC CAAACATATC
 -420 AGAGTAACCA TGACGAATAG TTTGTAGGAT ATCTTCTTGT CAAAAAGAAA AAAGAAAAAG
 -360 AAAAAGAAAT CTTCTTCTCC CATGCCAAAG TATGGAATCC CATAAACACC AATCACCATC
 -300 CTTTATTGCA ATGTTACCAA AACAGCCAAT AAAATAAAAT ATCTACTGAG AGGGAAATCT
 -240 AGCCCTCGAA TTCTCATTGG ATATCTAATG ATGATGTGGC AGATACTTTC TTTAAGATAA
 -180 TGTTATCCAC ACATCTTCTC TCTGATTTCT CTGGGGACCA CTCTCTCCCT TGTCCGAACT
 -120 TCACCTCCTC CACTCCTTCA CGAGTATACA TAAACGCGTA CACACTCTCG CAAAAATATC
  -60 AACTTCAGTA GTCTTCTTCA TCGCTATTGC TATCTCTCTC ACACTCTGTT TTGTCCAAAA
```

CCAAT motif Dof CAACA motif

C

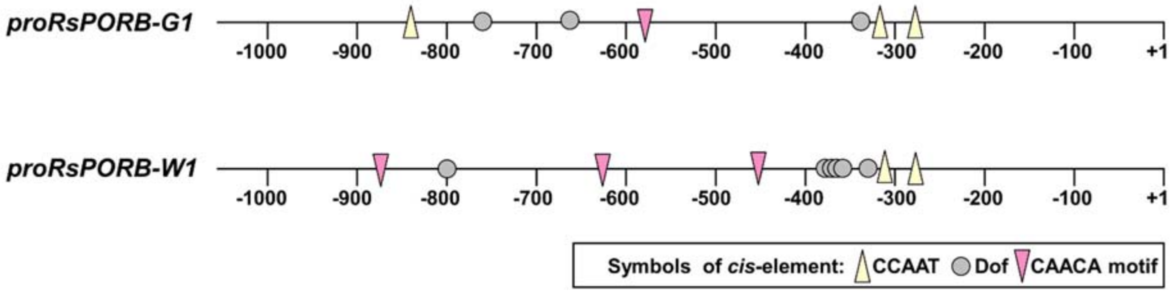

Figure S2. Nucleotide sequences of *RsPORB* promoters from radish cultivars G1 and W1. Partial promoter of *RsPORB-G1* (A) and *RsPORB-G1* (B). Putative several *cis*-elements are indicated by different colors. The CCAAT motif is indicated by a yellow box, Dof motif by dark grey boxes, and CAACA motif by pink boxes. (C) Schematic of the proRsPORB-G1 and proRsPORB-W1 showing putative cis-elements involved in chlorophyll biosynthesis. The *cis*-elements are indicated by different symbols.
